# Is Community-Led Total Sanitation connected to the rebuilding of latrines? Quantitative evidence from Mozambique

**DOI:** 10.1371/journal.pone.0197483

**Published:** 2018-05-22

**Authors:** Hans-Joachim Mosler, Sebastian Mosch, Miriam Harter

**Affiliations:** Department of Environmental Social Sciences, Swiss Federal Institute of Aquatic Science and Technology, Duebendorf, Switzerland; Public Library of Science, UNITED KINGDOM

## Abstract

To reduce open defecation, many implementers use the intervention strategies of Community-Led Total Sanitation (CLTS). But CLTS focuses on latrine construction and does not include latrine maintenance and repair damage or collapse. Some households rebuild their latrine while others return to open defecation. The reasons why are unknown. Using data from a cross-sectional survey, this article shows how physical, personal, and social context factors and psychosocial factors from the RANAS model are associated with CLTS participation, and how these factors connect to latrine rebuilding. In 2015, heavy rains hit the north of Mozambique and many latrines collapsed. Subsequently, 640 household interviews were conducted in the affected region. Logistic regression and mediation analyses reveal that latrine rebuilding depends on education, soil conditions, social cohesion, and a feeling of being safe from diarrhea, the perception that many other community members own a latrine, and high confidence in personal ability to repair or rebuild a broken latrine. The effect of CLTS is mediated through social and psychosocial factors. CLTS already targets most of the relevant factors, but can still be improved by including activities that would focus on other factors not yet sufficiently addressed.

## Introduction

The sixth of the Sustainable Development Goals set by the United Nations is to “ensure availability and sustainable management of water and sanitation for all”, but in 2015 some 2.4 billion people still lacked access to improved sanitation facilities [1]. This lack has fatal consequences: 6.3 million children died before their fifth birthday in 2013, and the corresponding figure predicted for 2030 is 4.4 million. Diarrheal diseases are amongst the first three reasons for child mortality in developing countries [2]. The use of latrines instead of defecating in the open could cut the prevalence of diarrheal diseases by up to 44% [3].

One answer to the problem of open defecation in rural areas in developing countries is Community-Led Total Sanitation (CLTS). This set of participatory and community-based activities successfully steers communities to change their behavior and become an open-defecation-free (ODF) community [4–6]. The commonly agreed goal of CLTS is for each household to own a latrine [7].

After some years of implementation, it is evident that CLTS is successful in engaging people in latrine construction and usage. However, CLTS has focused on building rather than on maintaining and repairing latrines in case of damage [8, 9]. It is not uncommon for people who were using latrines to abandon damaged, collapsed, and full pit latrines and return to open defecation [10–13].

Latrines collapse for a variety of reasons: sandy soil, natural disasters such as heavy floods, or termite attack [12, 14, 15]. Rain or floods may mean that people face the annual collapse of their latrines [16]. Some latrine users become discouraged and leave their broken or full latrines, while others do not [12]. Slippage (backsliding) rates of up to 92% have been reported [10]. However, one study in Malawi showed that up to 50% of those who had to deal with damaged latrines repaired them and started using them again [17]. Even poor people maintained their changed behavior and rebuilt broken latrines [18]. Which conditions lead people rebuild latrines or lead them to backslide to open defecation is unclear. Local structural, social, and individual parameters may account for peoples’ decisions. Whether individuals have to deal with difficult soil conditions or whether the surrounding community insists on remaining open defecation free can also influence rebuilding behavior. Whether the person feels demoralized by rebuilding a collapsed latrine or assesses the effort as very high may determine whether someone will reconstruct a latrine.

CLTS is effective in making people build latrines and use them [5, 19]. If CLTS is successful in changing lifelong habits, then it must somehow change some of the conditions that account for the decision to construct, and to reconstruct, a latrine. Of course, it cannot change relatively fixed conditions, such as soil conditions or income. But CLTS may influence how people assess those conditions. To achieve, CLTS alters something within people’s minds; it changes what are termed psychosocial factors in the mindsets of the people who are responsible for latrine building [20]. Is it possible that, by changing and maintaining these perceptions and attitudes, CLTS also influences rebuilding behavior? If we know how the success of CLTS in influencing rebuilding behavior is related to structural, social, and individual conditions, then we can improve CLTS so that it forms behavior that endures even after a challenge like latrine collapse.

To account theoretically for the structural, individual, and social conditions influencing latrine rebuilding, we combined the theory of triadic influence [21], which elaborates physical, personal, and social context factors with the risks, attitudes, norms, abilities, and self-regulation (RANAS) model of behavior change. The result is a comprehensive framework which incorporates both the influences of physical, personal, and social context factors and the psychosocial factors of the RANAS model.

The physical context includes information about natural and built conditions. Both soil conditions and reasons for the collapse of latrines are taken into account.

Socio-demographic characteristics such as age, sex, education, income, household size, and religion form part of the personal context, as do the physical abilities of the individual.

The social context is formed by broad parameters such as culture, laws, policies, and the information available (e.g. CLTS events). In steering a community towards becoming and remaining open defecation free (ODF), CLTS needs to concentrate “on the whole community rather than on individual behaviors” [7]. The interaction of community members and the social environment is crucial for the success of CLTS [22]. Therefore, the concepts of social capital, social dilemma and social identity need to be considered. The construct of social capital groups together such factors as trust, cooperation, communication, and social cohesion [23]. Social dilemma describes situations in which an individual is better off when not behaving in a way that benefits the whole group. Latrine construction can be seen as such: one can save costs and the effort of maintenance by defecating in the open, but this creates a risk for the whole community [24–26]. Social identity describes how individuals are connected within the group and how strong the in-group ties are [27]. These are also important factors in explaining actions towards a common goal.

Besides the personal, physical, and social context factors, psychosocial factors are part of the framework: within the RANAS model, psychosocial factors are grouped in five blocks. The first block comprises the risk factors, which represent a person’s understanding and awareness of health risks. Attitude factors appear in the second block. They are a person’s positive or negative stance towards a behavior. Norm factors form the third block; they represent the perceived social pressure towards that behavior. The ability factors form the fourth block. They denote a person’s confidence in her or his ability to practice the behavior. Self-regulation factors form the last block. They represent a person’s attempts to plan and self-monitor their performance of the behavior and to manage conflicting goals and distracting cues.

This study investigates the effects of CLTS participation on latrine rebuilding and the influences of CLTS participation on personal, physical, and social context factors and psychosocial factors by conducting a cross-sectional survey in Mozambique. Latrine rebuilders are compared to non-rebuilders, and CLTS participation is compared to non-participation. The survey was conducted in 2015, a maximum of 8 months after a CLTS implementation by Pathfinder International in northern Mozambique. CLTS was implemented according to the official CLTS handbook (7) and followed three phases of implementation. Pre-triggering involves the evaluation of pre-existing social and physical conditions so that the triggering activities can be adapted to them. Triggering activities are implemented at a community meeting and encourage collective action towards an open-defecation-free environment. The actions that generate such an environment are afterwards monitored during the post-triggering phase.

In early 2015, heavy rains hit the north of Mozambique. The resultant floods destroyed 10,860 houses (Mozambique: Floods Emergency Appeal MDRMZ011 Final Report: International Federation of Red Cross and Red Crescent Societies, 2015). Before the rains, the northern district of Nampula was chosen for the survey. Several of the communities in the province to be surveyed had been declared as ODF. However, by the time the survey was conducted, many latrines had collapsed, and the status of the communities could no longer be defined.

In summary, this paper addresses two research questions:

Which structural, personal, and social context factors and psychosocial factors favor or hamper latrine rebuilding?

Through which social context factors and psychosocial factors is CLTS associated with latrine rebuilding?

## Methods

### Research area and participants

The data were collected in the northern region of Mozambique, namely in the rural communities of Nampula district. Two criteria were used for the selection of the communities: (1) only those communities where SCIP Nampula (a program implemented by Pathfinder International and partners, funded by USAID) had realized CLTS in the past 8 months were eligible and (2) communities should have comprised of more than 20 households. Of the communities meeting the two criteria, a list of 26 communities were selected randomly.

A team of 10 local data collectors was selected and trained. The training included the discussion of the questionnaire, underlying psychological concepts and interviewing guidelines, role-playing the interviews, and detailed discussions of ethical considerations. The data collectors were accompanied by two study managers and one local field coordinator. Within the communities, 20 households were randomly selected using a modified random route method based on Hoffmeyer-Zlotnik [28]. With the help of community members, the structure of the communities was discussed, and every data collector was assigned to one section of the community. In this section, he or she selected every third household. Individuals chiefly responsible for child care and food preparation were selected as respondents. They were believed to have most insight into daily defecation practices of all family members and the hygiene situation of the household. The interviews lasted approximately one hour.

### Sample

The sample is spread over four districts, with 6.8% located in Meconta, 29.9% in Angoche, 32.4% in Monapo, and 30.9% in Mogovolas. A total of 22 households refused to participate, and another 292 households were not interviewed because no one was at home or the individual present did not fit the selection criteria (final sample: 640 households). From this sample, a subsample of 288 households was selected for the analyses presented in this paper. Households were selected only if they had had a latrine at one point but stopped using it due to latrine damage or collapse. Households that had returned to the practice of open defecation were compared to households that had rebuilt their latrines. A total of 10 cases were excluded due to missing data (final sample size: 278). Details of the subsample are described in Table 1.

**Table 1:** Characteristics of the sample.

	comparison group		
characteristics of respondent and household	no rebuilding	rebuilding	*p* value
female respondent	99.3	98.7	0.537
age	34.3	36.2	0.238
Muslim	38.3%	51.7%	0.154
cohabiting	59.1%	60.3%	0.788
years of education	1.9	2.7	0.003
ability to read and write	8.8%	18.5%	0.012
farming	85.8%	81.5%	0.558
monthly income	670.5	761.1	0.485
owns house	98.5%	98.0%	0.54
household size	4.9	5.3	0.122
number of children <5	1.0	1.1	0.5
dirty household surrounding	33.6%	38.7%	0.22
latrine use	10.2%	93.4%	<0.001
*open defecation*	*85*.*4%*	*0*.*7%*	
*mixed user*	*4*.*4%*	*6*.*0%*	
distance to od area (> 20 minutes)	9.50%	25.20%	<0.001
rocky soil	12.60%	3.30%	<0.001
participation in CLTS	37.2%	62.3%	<0.001

All data is self-reported at the point after CLTS-intervention. A t-test or chi-square test was used to check for significant differences.

### Questionnaire and measures

A structured questionnaire was designed based on the RANAS model [20]. It included questions about the socio-demographic characteristics of the household and psychosocial factors potentially steering latrine construction, latrine use and open defecation. Further questions were incorporated to assess the CLTS interventions and subsidy policy. Lastly, items assessing social capital, social cohesion, and social dilemma were included. The items were answered using 5-point scales for unipolar items and 7-point scales for bipolar questions; all answers were self-reported. The questionnaire was first drafted in English, then translated into Portuguese and discussed with the data collectors in the local language, Makhuwa. The questionnaire was pretested in 20 interviews under real conditions and adapted where necessary.

### Independent and dependent variables

CLTS participation: CLTS intervention was used as dichotomous primary independent variable in a multiple mediation analysis. People who participated in CLTS activities (e.g., the triggering event) and people who did not participate but received CLTS related information indirectly from relatives, friends, or neighbors formed the group of CLTS receivers (self-reported). This group was compared to the group of CLTS non-receivers, who neither participated in CLTS activities nor received CLTS-related information from others. CLTS receivers were coded ‘1’, and non-receivers were coded ‘0’.

Latrine rebuilding: Latrine rebuilding was used as dichotomous dependent variable in four logistic regression models and in multiple mediation analysis and is based on self-reported latrine rebuilding after latrine use stoppage. Latrine rebuilders were coded ‘1’, and non-rebuilders were coded ‘0’.

Contextual and psychosocial factors: Several context factors were used as independent variables in logistic regression analysis (Table 2).

**Table 2:** Personal, physical and social context factors.

Personal context	Physical context	Social context
Age	Risk of flooding	Social dilemma
Relationship status	Soil conditions	Social capital
Years at school		Social identity
Ability to read/ write		Social cohesion
Religion		
Household size		
Average monthly income/family		

Relationship status and ability to read and write were dichotomous items that distinguished people in a relationship from those not in a relationship and individuals who were able to read and write from those not able to read or write. Religion of respondents was categorized as Muslim, Catholic, and tribal or other religions. Soil conditions are classified as sandy, clayey, or rocky. Continuous scales were used for age, years at school, household size, average monthly income, risk of flooding, and all the social context factors (details of the measurements of these factors are displayed in Supporting Information S1 Table). The psychosocial determinants of the RANAS model were assessed using questions that framed latrine construction as a target behavior (for details of the questions see Supporting Information S2 Table).

### Data analysis procedure

To answer the first research question (“Which structural, personal, and social context factors and psychosocial factors favour or hamper latrine rebuilding?”), four logistic regression models were performed to identify contextual and psychosocial factors which promised to be relevant in explaining latrine rebuilding. The first model tested for personal and physical context factors; the second tested for social context factors, and the third tested for psychosocial factors. In the fourth logistic regression model, significant factors from the first three models were included to determine which factors were crucial in predicting latrine rebuilding.

To answer the second research question (“Through which social context factors and psychosocial factors is CLTS associated with latrine rebuilding?”), a multiple mediation analysis was conducted to identify the social context factors and psychosocial factors that associated CLTS with latrine rebuilding. To do this, all statistically significant social context and psychosocial factors from the fourth logistic regression model were included as mediators in this analysis. The binary outcome variable distinguishes between people with and people without a rebuilt latrine, and the dichotomous independent variable discriminates between CLTS receivers and non-receivers. The multiple mediation analysis was performed using PROCESS for SPSS [29]. The data analysis was carried out using SPSS version 22.

### Ethical considerations

Oral informed consent was obtained from each participant, and information was given about the background of the study, the voluntary nature of participation, and the anonymous usage of data. The research procedures and measurements were reviewed and approved by the ethics board at the Faculty of Arts of the University of Zurich and the National Committee of Bioethics for Health Mozambique (CNBS) and strictly followed ethical principles of the American Psychological Association (APA) and the Declaration of Helsinki.

## Results

### Demographics

**Fig 1:**
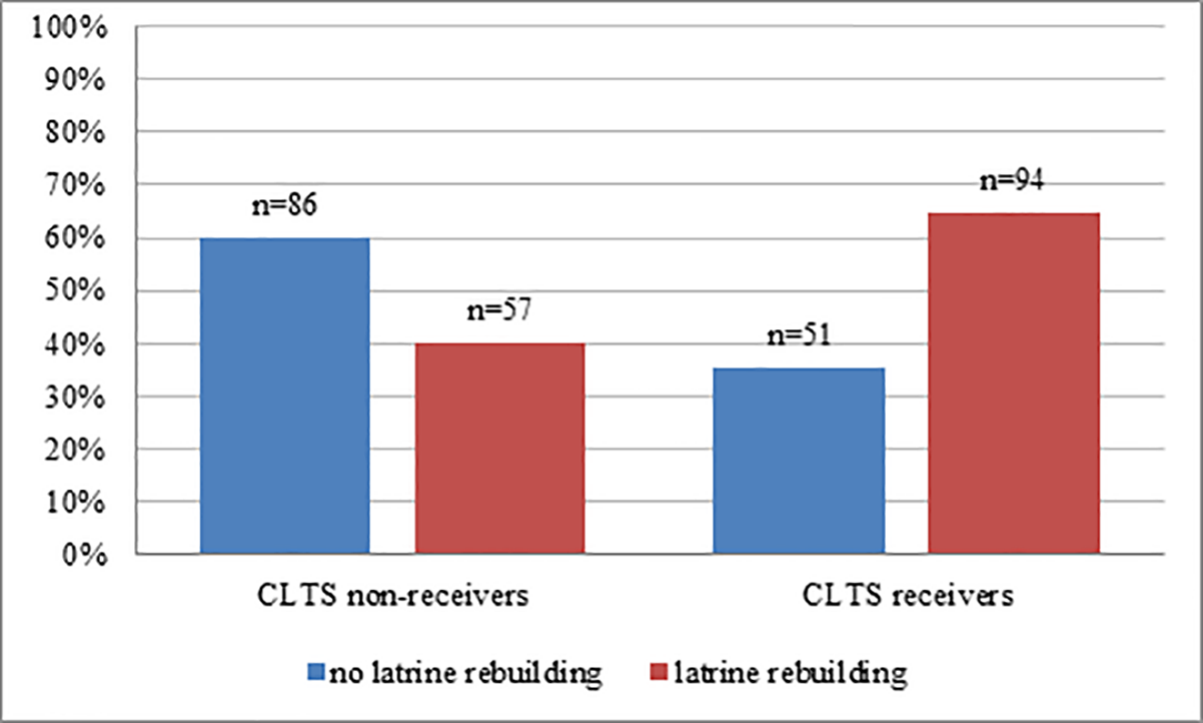
Effect of CLTS on rebuilding. Differences in latrine rebuilding between people who received CLTS-related information and people who did not.

The sample consisted of 99.5% female respondents; the mean age was 35 years (SD = 15.5). Of all the respondents, 80.6% reported that they were in a relationship. The respondents had attended school for 2.3 years on average (SD = 2.4); 13.9% were able to read and write. Some 43.8% were Muslims, 49% were Catholic, and 7.2% belonged to tribal religions or others. The average household contained five members (SD = 1.9), and the mean income was MZN 1,406.20 (approx. USD 18.25, exchange rate 31.10.2016) per household (SD = USD 30) per month. Of those households observed to own a latrine, 87.3% also reported using it daily, 12.3% irregularly or never. Of all respondents, 49.8% indicated that their village is not at all subject to flooding, 22.3% reported their village is somewhat at risk of flooding, 10.8% said rather at risk, 12.5% estimated the risk as quite high, and 4.6% indicated that their village is very much subject to flooding. Half of the sample, 50.3%, received CLTS-related information and were therefore classified as CLTS-receivers, while 49.7% were classified as non-receivers. The association between latrine rebuilding and CLTS reception was statistically significant, χ2(1) = 17.995, ***p*** < .0005, with all expected cell frequencies being greater than five. Of those who received CLTS, 64.8% rebuilt their latrine; the equivalent figure for CLTS non-receivers was 39.9% (Fig 1).

### Reasons for collapse and rebuilding

**Fig 2:**
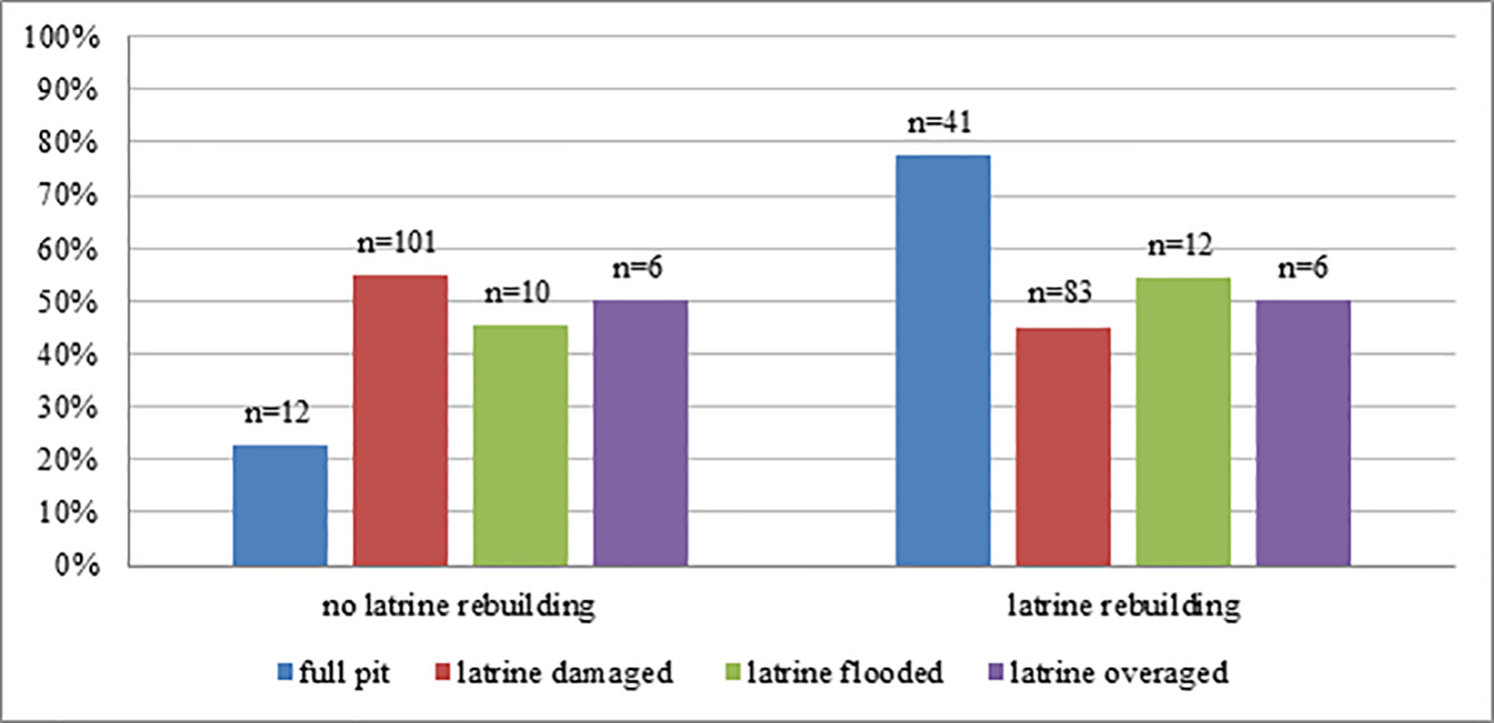
Latrine decommission and latrine rebuilding. Differences in latrine rebuilding related to the reported cause of latrine decommission.

Most of the observed latrines were simple pit latrines (94.4%), built of local materials such as mud and straw; only 5.4% were improved pit latrines with a squat plate made of cement and 0.2% were pour flush latrines connected to a pit. Latrine stoppages occurred frequently in the sample in Mozambique. In total, 47.8% (n = 288) of all respondents reported having been in possession of a latrine in the past and that their latrine had been out of use at least once. Of this group, 52.6% had repaired their latrines and were currently using it. The main reasons for latrines being out of use were damage (67.9%) and a full pit (19.6%). These were followed by flooding due to rainfalls (8.1%), and 4.4% of the latrines were classified as too old by their owners and were out of order as a result. Latrines that were out of use because they were full were most likely to be brought back into use (77.4%), but less than half of those that had been damaged were repaired (45.1%) (Fig 2).

**Fig 3:**
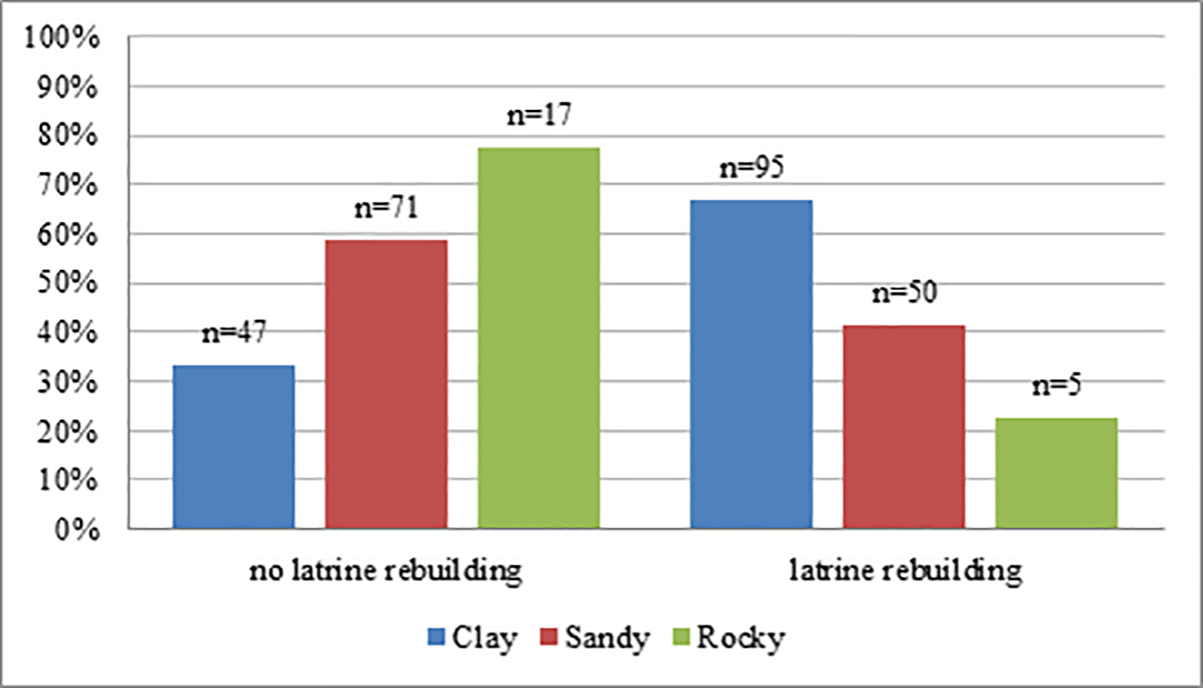
Soil conditions and latrine rebuilding. Differences in latrine rebuilding related to reported soil conditions by respondents. Clayey = clayey soil; sandy = sandy soil; rocky = rocky soil.

Almost half of the respondents (49.8%) lived in areas with clayey soil, 42.5% reported living in areas with sandy soil, and 7.7% with rocky soil. Of all people living in areas with clayey soil, 66.9% rebuilt their latrines, 41.3% of people with sandy soil rebuilt their latrine, and only 22.7% of people with rocky soil rebuilt them (Fig 3).

**Fig 4:**
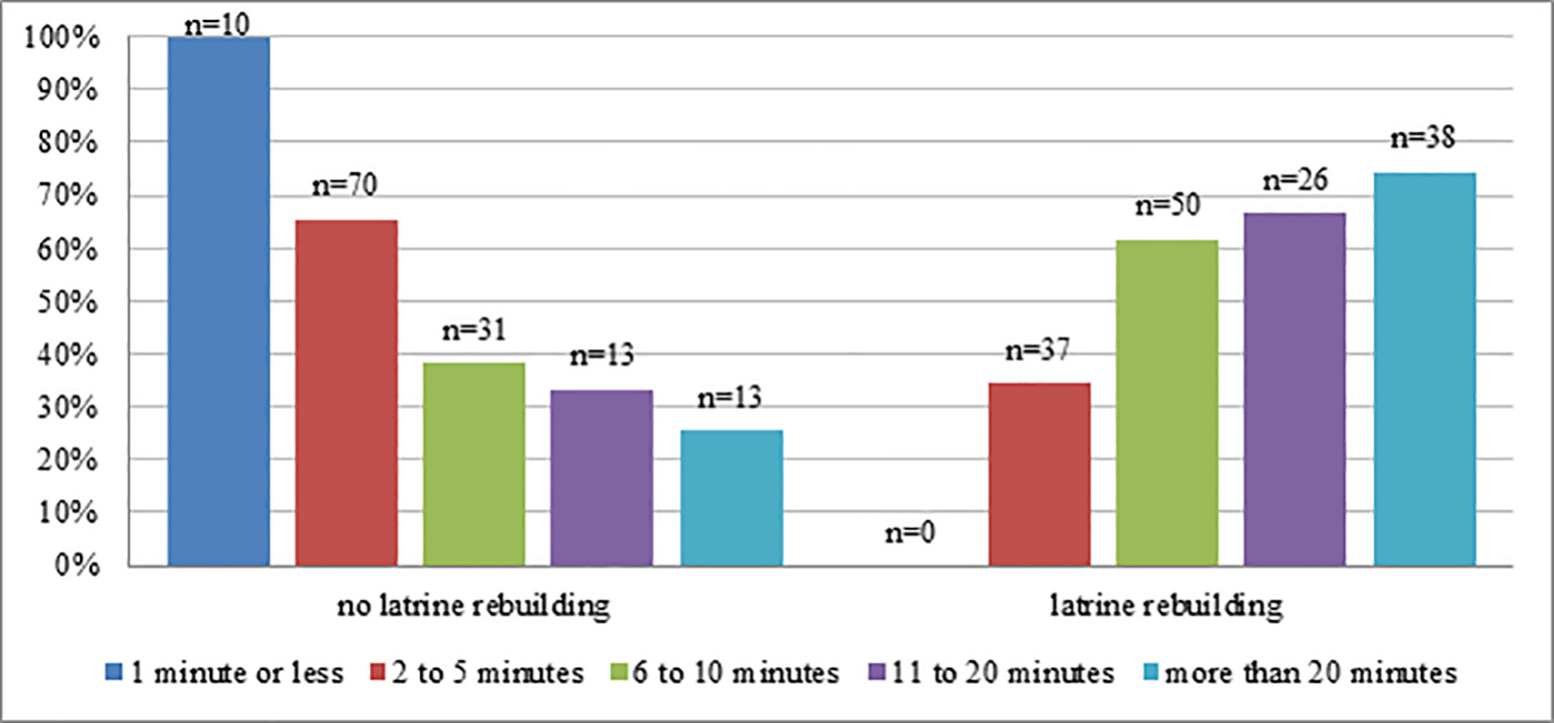
Distance to OD area and latrine rebuilding. Differences in latrine rebuilding related to self-reported walking distance to the nearest potential open defecation area.

Furthermore, people seem to rebuild their latrines more often when a potential place for open defecation is farther away. For example, 74.5% of the individuals living more than 20 minutes away from an open defecation area rebuilt their latrines, but no one rebuilt a latrine if a place for open defecation was within a minute’s walk (Fig 4).

### Research question 1

To answer the first research question, about which structural, personal, and social context factors and psychosocial factors favor or hamper latrine rebuilding, four logistic regressions were performed to determine the effects of context and psychosocial factors on the likelihood that participants rebuild their latrine in case of damage or collapse. The linearity of the continuous variables of all four regressions was assessed using the Box-Tidwell [30] procedure; all continuous independent variables were found to be linearly related to the logit of the dependent variable. All regression models were checked for multicollinearity and variance-inflation-factors (VIF) found to be unproblematic according to Myers [31].

The first regression model estimates the influence of personal and physical context factors on the probability that people rebuild their latrine (Supporting Information S3 Table). The logistic regression model was statistically significant, χ^2^(12) = 82.344, *p* < .005. The model explained 35.3% (Nagelkerke *R*^*2*^) of the variance in latrine rebuilding and correctly classified 76% of cases. Of all nine predictor variables, four context factors remained statistically significant: years at school, risk of flooding, rocky soil, and distance to OD area (as shown in Table 2). More years at school and living in areas with reduced risk of flooding were associated with latrine rebuilding. People living in areas with primarily rocky soil were 0.23 times more likely to rebuild a latrine than people living in areas with primarily sandy soil. People having a larger distance to the OD area were 2.06 times more likely to rebuild their latrine.

The second regression model estimates the influence of social context factors on the probability that people rebuild their latrine (Supporting Information S3 Table). The logistic regression model was statistically significant, χ^2^(10) = 65.11, *p* < .0005. The model explained 26.7% (Nagelkerke *R*^*2*^) of the variance in latrine rebuilding and correctly classified 72.5% of cases. Of all ten social context factors, five remained statistically significant and are associated with latrine rebuilding: social dilemma, solidarity (social capital), trust (social capital), social cohesion and inclusion (social capital), and in-group ties (social identity). Living in communities with a higher collective ambition to reduce open defecation, a stronger solidarity within the village, higher trust between the residents, a stronger sense of cohesion and inclusion within the village, and stronger in-group ties are all associated with latrine rebuilding.

The third regression model determines psychosocial factors and their association with latrine rebuilding (Supporting Information S3 Table). The logistic regression model was statistically significant, χ^2^(21) = 198.444, *p* < .0005. The model explained 68.2% (Nagelkerke *R*^*2*^) of the variance in latrine rebuilding and correctly classified 85.6% of cases. In total, five psychosocial factors remain significant: personal general risk of diarrhea (vulnerability), general health of community members (vulnerability), others’ behavior (community members), confidence in recovery, and communication. Feeling less vulnerable to diarrhea and estimating the health risk for community members as higher because of open defecation are both associated with latrine rebuilding. Furthermore, a higher estimation of the number of latrine owners in the community (others’ behavior), higher confidence in being able to rebuild the latrine in case of damage or collapse, and talking about latrine-related topics more often are also associated with latrine rebuilding.

In the fourth and final regression model (Table 3), significant context and psychosocial factors from the first three models were combined to determine the factors relevant to latrine rebuilding. The logistic regression model was statistically significant, χ^2^(16) = 198.247, *p* < .0005. The model explained 68% (Nagelkerke *R*^*2*^) of the variance in latrine rebuilding and correctly classified 84.9% of cases. On combining all factors, risk of flooding, from the personal and physical context factor block, lost its significant influence on the likelihood of latrine rebuilding, as did social dilemma, social capital (trust and solidarity), and social identity (in-group ties) from the social context factors block. Significant associations remained between latrine rebuilding and years at school, soil conditions, and social capital (social cohesion and inclusion). All factors that were detected in the third model for the psychosocial factor block, except for communication, maintained their significance in distinguishing between latrine rebuilders and non-rebuilders. More years at school and living in communities with a stronger sense of social cohesion are associated with latrine rebuilding. People living in areas with clayey soil are 2.39 times more likely to rebuild their latrines than people living in areas with sandy soil, and people living in areas with rocky soil are 3.29 times less likely to rebuild. Feeling less vulnerable to diarrhea and estimating the health risk for community members as higher because of one’s own open defecation behavior is connected with latrine rebuilding, as is a higher estimation of the number of latrine owners within the community (others’ behaviour) and a higher confidence in being able to rebuild the latrine in case of damage or collapse.

**Table 3:** Predictors of latrine rebuilding in logistic regression analysis.

Modell	B	SE	Wald X^2^ (1)	OR	95% CI
**Modell 4: significant context and RANAS factors from model 1+2+3**					
**Context factors**					
Relationship status^a^	.741	.501	2.189	2.09	.78, 5.59
Years at school	.250	.089	7.846**	1.28	1.07, 1.53
Risk of flooding	-.099	.168	.344	.90	.65, 1.26
Soil condition: Clay^d^	.873	.436	4.014*	2.39	1.01, 5.62
Soil condition: Rocky^d^	-1.600	.881	3.299†	.20	.03, 1.13
Social dilemma	.039	.177	.048	1.03	.73, 1.46
Social capital (solidarity)	.136	.127	1.158	1.14	.89, 1.46
Social capital (trust)	-.044	.140	.098	.95	.72, 1.25
Social capital (social cohesion and inclusion)	.445	.160	7.753**	1.56	1.14, 2.13
Social identity (in-group ties)	.101	.143	.503	1.10	.83, 1.46
Distance to OD area	.265	.180	2.163	1.30	.91, 1.85
**RANAS factors**					
Vulnerability (personal general risk for diarrhea)	-.611	.155	15.508***	.54	.40, .73
Vulnerability (general health of community members)	.657	.340	3.735†	1.92	.99, 3.75
Estimated number of other latrine owners (Others’ behavior/ community members)	1.106	.221	24.938***	3.02	1.95, 4.66
Confidence in recovery	.910	.271	11.321**	2.48	1.46, 4.22
Communication	.118	.201	.343	1.12	.75, 1.66
Constant	-13.273	2.545	27.208***		

*N* = 278. For the overall model of significant context and psychosocial factors (Model 4) R^2^ = .68 (Nagelkerke). X^2^(16) =, p < .0005. OR = odds ratio; CI = confidence interval

^a^no relationship as reference category

^d^ sandy as reference category

*P = .05

**P = .005

***P = .0005

† P < .06

### Research question 2

**Fig 5:**
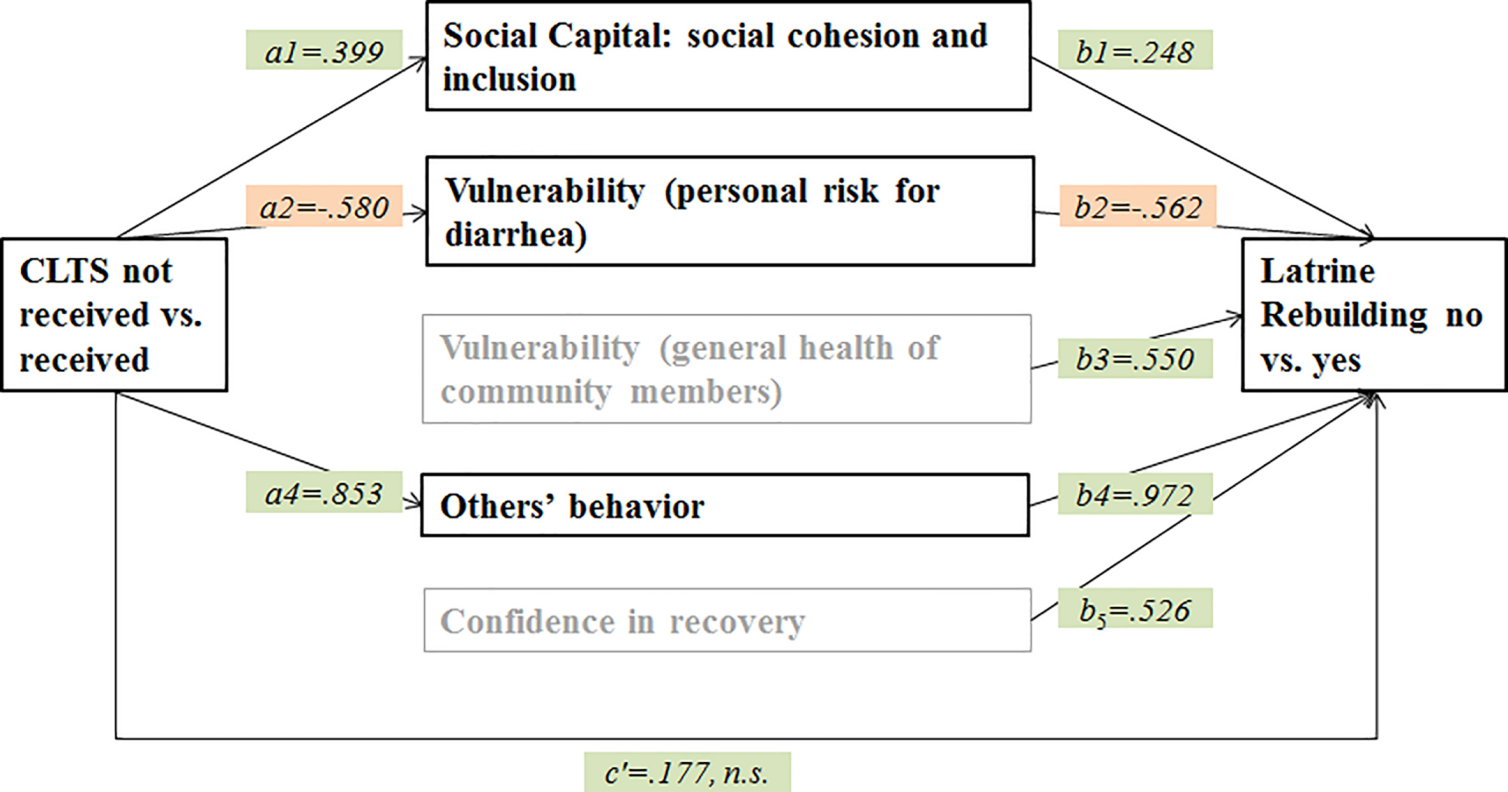
Statistical diagram of the multiple mediation model for the indirect influence of CLTS on latrine rebuilding through several social and psycho-social context factors. CLTS received was coded ‘1’ and CLTS not received was coded ‘0’. Latrine rebuilding was coded ‘1’ and no latrine rebuilding was coded ‘0’. a1 –a4 = unstandardized regression coefficients from linear regressions; b1 –b5 = unstandardized regression coefficients from logistic regression; c’ = indirect effect of CLTS on latrine ownership status.

For the second research question, through which personal and social context factors and psychosocial factors is CLTS associated with latrine rebuilding, social and psychosocial factors identified in the logistic regressions were used as mediators in a multiple mediation analysis. Structural and personal context factors were not included, because of their unchanging nature. As can be seen in Table 4, CLTS indirectly influenced the probability of latrine rebuilding through its effects on social cohesion and inclusion within the community (OR = 1.104), the estimation of personal risk of diarrhea (OR = 1.385), and the behavior of other community members (OR = 2.291). As can be seen in Fig 5 and Table 4, in comparison to people that did not receive CLTS, participants that received CLTS felt a stronger sense of cohesion and inclusion within their communities (*a*_1_ = .399), felt less vulnerable to diarrhea (*a*_2_ = -.580), and estimated the number of latrine owners (others’ behaviour) within their community as higher (*a*_4_ = .853). Latrine rebuilding is associated with a stronger sense of cohesion and inclusion within the community (*b*_1_ = .248), lower perceived vulnerability to diarrhea (*b*_2_ = -.562), and a higher estimation of the number of latrine owners (others’ behaviour) within the community (*b*_4_ = 0.972). As Fig 5 shows, people who expressed a stronger conviction that their personal defecation behavior affects the health of other community members are more likely to rebuild their latrine, as are people who felt more confident in being able to rebuild their latrine in case of damage or collapse. However, the analysis showed that these factors are not significantly influenced by the CLTS intervention. Bias-corrected bootstrap intervals based on 10,000 bootstrap samples and odds ratios have been computed for all specific indirect effects (see Table 4). There was no evidence that CLTS influenced the probability of latrine rebuilding independently of its effect on the factors mentioned above (*c’* = .177, *p* = .593). People who participated in CLTS reported higher cohesion within their communities and higher density of latrine owners in their surroundings. These two characteristics are positively connected to latrine rebuilding. Participants also assessed their risk of becoming infected with diarrhea as lower than non-participants; people with a lower risk perception are more likely to rebuild an out-of-use latrine. Feeling more confident in being able to repair a broken latrine and assessing the health risk for the community as higher when personally defecating in the open are both connected with higher latrine rebuilding likelihood, but these factors do not differ between CLTS participants and non-participants.

**Table 4:** Summary of multiple mediation analysis.

Mediator	CLTS			Latrine rebuilding			Indirect effect (95% CI)			Odds ratio for specific indirect effects (95% CI)
	*B*	*SE*	*p*	*B*	*SE*	*p*	LL	*B*	UL	OR
**Social capital (social cohesion and inclusion)**	0.399	0.163	.015	0.248	0.119	.037	0.006	0.099	0.284	**1.104**
**Vulnerability (personal general risk for diarrhea)**	-0.580	0.161	.000	-0.562	0.125	.000	0.125	0.326	0.629	**1.385**
**Vulnerability (general health of community members)**	0.042	0.076	.582	0.550	0.260	.034	-0.057	0.023	0.156	1.023
**Others’ behavior (Estimated number of other latrine owners)**	0.853	0.139	.000	0.972	0.159	.000	0.475	0.829	1.294	**2.291**
**Confidence in recovery**	0.049	0.109	.653	0.526	0.191	.006	-0.082	0.026	0.203	1.026

CLTS Indirectly Influencing Latrine Rebuilding through its Effect on Several Social and Psychosocial Factors. N = 286. B = unstandardized regression coefficients from linear regressions (CLTS) and logistic regression (latrine ownership); SE = standard error; CI = confidence interval for specific indirect effects; LL = lower limit; UL = upper limit; OR = odds ratio for specific indirect effects. CLTS received was coded ‘1’ and CLTS not received was coded ‘0’; latrine rebuilding was coded ‘1’ and no latrine rebuilding was coded ‘0’. Bias-corrected bootstrap confidence intervals for the specific indirect effects has been computed based on 10,000 bootstrap samples (bold: Significant effects).

## Discussion

The results presented here indicate that rebuilding of decommissioned latrines is related to a) favorable personal and physical context factors such as education and soil conditions, b) CLTS strengthening the social structure and perceived latrine ownership of others within the community, and c) low risk perception by people owning and maintaining latrines, which is supported by information received during CLTS interventions.

The reasons for latrines being out of use are diverse but cannot account for rebuilding behavior, as people either rebuilt or didn’t rebuild latrines that were out of use for every one of the reasons mentioned. If the pit is full or if it is flooded, some people recommission what they used to have. This study shows that other reasons can better explain why people rebuild their latrines, such as social and psychosocial factors. Perhaps predictably, people who have to walk longer distances to open defecation areas are more likely to rebuild latrines. The effort involved in open defecation could be a future argument to focus on during CLTS interventions. With growing population density, finding suitable places will become more problematic. A more difficult result to address is that people living in rocky areas show lower rebuilding behavior than people in other areas. The question arises how successful CLTS can be in areas where digging a pit is very effortful. But the mediation analysis showed, confidence in being able to repair a broken latrine could solve this difficulty. As former research has already shown, education is a factor explaining the success of CLTS [32, 33] as well as latrine ownership [34]. This study also showed that the number of years in school is important for latrine rebuilding. CLTS implementers should be aware of the importance of community members with higher levels of education, as they could be the ones taking the lead in CLTS activities.

The regression models support earlier results indicating that rebuilding cannot be explained solely by income or by difficult physical context conditions [18]: a strong feeling of being connected with other community members and favorable psychosocial factors is also associated to latrine rebuilding. Other authors have already emphasized the importance of social processes for the success of CLTS [8, 35], but their focus has primarily been latrine construction rather than repair. This study showed that a positive feeling of cohesion and inclusion is closely related to the probability of rebuilding. During the pre-triggering process, communities are usually assessed to gain an understanding of the preconditions for CLTS. During this process, communities’ social cohesion and inclusion should be investigated and if necessary targeted during the triggering session. For latrine rebuilding, it is also important that people perceive themselves as surrounded by other latrine owners. This result is in line with previous research showing that the number of others performing a behavior helps to account for one’s own behavior [36–39].

Rebuilding is also connected with the belief that the individual’s own risk of becoming infected with diarrhea is lower when using a latrine. This could be because latrine users feel hygienic and safe from coming into contact with bacteria as also shown by former research on drivers for latrine construction [34]To keep this standard, they also put more effort into keeping the latrine and are more likely to repair or rebuild it if necessary.

The mediation analysis also reveals that the CLTS participation is associated to latrine rebuilding together with corresponding changes to the mindsets of the participants. What has been demonstrated by other research projects using the RANAS approach of behavior change [37, 40, 41] is replicated in this project: behavior change involves changes within the person that receives the information. Furthermore, CLTS is already very successful in targeting important factors for latrine rebuilding, such as strengthening social cohesion and inclusion and informing people about their personal risk of diarrhea and the perceived quantity of people owning and using a latrine in their surroundings. But it can still be improved as well. The analysis showed that, of five psychosocial factors that help to account for latrine rebuilding, only three are changed by CLTS participation. The addition of data-based behavior change strategies could improve effectiveness even more.

Not only is it important to assess one’s own risk of becoming infected with diarrhea but also the risk for the whole community. People should think about their responsibility when defecating in the open. If this awareness is strong, people show higher levels of rebuilding behavior. Behavior change techniques could be included to target this factor in particular, as described in the catalog of behavior change techniques of the RANAS approach [42].

For example, during open defecation mapping, which is usually performed during the pre-triggering phase, the fact could be highlighted that even a single person defecating in the open affects the health of all other members of the community. After the community session, personal health risks could be assessed in household visits, and again the effect of others’ behavior on personal risk could be discussed individually. This accounts for the phenomenon known as collective action or social dilemma [25].

Secondly, people need to have strong confidence in their own ability to repair or rebuild a broken latrine; then they are more likely to do so. CLTS still lacks effective means to support and strengthen this belief. This could be achieved by discussing with community members the fact that latrines may be put out of use by external factors. The important message is that this is not a sign of failure and should not be considered a reflection of one’s own capability. Instead of being discouraged, people could find social support, repair or rebuild their latrine, and start using it again. The importance of maintaining the new behavior and not backsliding to open defecation should be highlighted.

### Limitations of this study

Data for these analyses is all self-reported by respondents and was collected in a cross-sectional survey. This does not allow causal inferences to be drawn. Furthermore, the analyses should be repeated with a larger sample size to account for the rather large amount of predictors included in the regression analyses. However, this is the first study to our knowledge that assesses the reasons for rebuilding and their connections to CLTS taking into account not only physical context conditions but also social and psychosocial factors. This study therefore reveals that, apart from soil conditions and years of school, three other factors–strong social cohesion, a detailed assessment of the risk of diarrhea and the perception of others’ latrine usage–help to account for latrine rebuilding and are successfully targeted by CLTS. Two further factors are identified that are important for latrine rebuilding but are not sufficiently addressed by CLTS.

### Indications for practice and future research

When a CLTS intervention is planned, the results of this study should be taken into account and integrated into the set of activities, especially in areas where latrines can be expected to be out of use. The long-term goals of both latrine construction and latrine maintenance could be better achieved by including the behavior change techniques identified in this study. Targeting the awareness of the risk for the whole community of getting diarrhea and the belief to be able to repair or rebuild a broken latrine could help implementing organizations to act more efficiently and effectively.

Future research should include observations of behavior and include longitudinal data for a deeper understanding of processes that make CLTS successful. Changes in psychosocial factors could be measured as in this paper and compared with latrine rebuilding behavior before and after a CLTS implementation. The research group is currently addressing such work in a CLTS implementation in Ghana.

## Supporting information

S1 Table Items and answer categories for personal, physical and social context factors. (DOCX)

S2 Table Items and answer categories for psychosocial factors (RANAS). (DOCX)

S3 Table Predictors of latrine rebuilding in logistic regression analysis. (DOCX)
