# Supplementary material for: Is Community-Led Total Sanitation connected to the rebuilding of latrines? Quantitative evidence from Mozambique
Source: PLoS One. 2018 May 22;13(5):e0197483. doi: 10.1371/journal.pone.0197483 (PMC5963780; doi:10.1371/journal.pone.0197483)
Supplement: S1 Table — (DOCX) [file pone.0197483.s001.docx]

**S1 Table.** **Items and Answer Categories for Personal, Physical and Social Context Factors.**

| **Factor** | **Item example** | **Answer category** |
| --- | --- | --- |
| **Personal and physical factors** | | |
| **Age** | What is your age? | Open question |
| **Relationship status** | What is your relationship status? | Open question |
| **Years at school** | How many years did you go to school? | Open question |
| **Able to read/ write** | Are you able to read/ write? | Open question |
| **Religion** | What is your religion? | Open question |
| **Household size** | How many people live in this household in total? | Open question |
| **Household income** | What is the average monthly income of your family in Meticais (MZN)? | Open question |
| **Risk of flooding** | Are any parts of the village subject to flooding? | 1=not at all subject to flooding to  2=very much subject to flooding |
| **Soil conditions** | What are the general soil conditions like in the village? | Open question |
| **Distance to OD area** | How many minutes does it take from your house to get to the place where you defecate openly? | 1=1 minute or less to  5=more than 20 minutes |
| **Social factors** | | |
| **Social dilemma** | How much do you feel your community is working together in reducing open defecation? | 1=not at all to  5=very much |
| **Social capital: Solidarity** | If a community project does not directly benefit me but has benefits for many others in the community, I would contribute time or money to the project. | 1=extremely disagree to 7=extremely agree |
| **Social capital: Trust** | Most people who live in this community can be trusted. | 1=extremely disagree to 7=extremely agree |
| **Social Capital: Empowerment and political action** | I have the freedom to make important decisions that change my life. | 1=extremely disagree to 7=extremely agree |
| **Social capital: Collective Action and Cooperation** | If there is a sanitation problem in this community, how likely is it that people will cooperate to try to solve the problem? | 1=not at all likely to 5=very likely |
| **Social Capital: Social cohesion and inclusion** | In the last month, how many times have you met with people in a public place either to talk or to have food or drinks? | 1=never  2=always |
| **Social Identity: In-group Ties** | I have a lot in common with other community members. | 1=extremely disagree to 7=extremely agree |
| **Social Identity: Centrality** | In general, being a member of this community is an important part of my self-image. | 1=extremely disagree to 7=extremely agree |
| **Social Identity: In-group Affects** | In general, I’m glad to be a member of this community. | 1=extremely disagree to 7=extremely agree |
| **Social cohesion: Neighborhood cohesion** | I would be willing to work together with others on something to improve my community. | 1=extremely disagree to 7=extremely agree |
