# Supplementary material for: Is Community-Led Total Sanitation connected to the rebuilding of latrines? Quantitative evidence from Mozambique
Source: PLoS One. 2018 May 22;13(5):e0197483. doi: 10.1371/journal.pone.0197483 (PMC5963780; doi:10.1371/journal.pone.0197483)
Supplement: S2 Table — (DOCX) [file pone.0197483.s002.docx]

**S2 Table. Items and Answer Categories for Psychosocial Factors (RANAS).**

| **Factor** | **Item example** | **Answer category** |
| --- | --- | --- |
| Vulnerability (personal general risk for diarrhea) | Generally, how high do you think is the risk that you get diarrhea? | 1=not at all high to  5=very high |
| Vulnerability (general health of community members) | If you defecate in the open, does this affect the health of other members of the community? | 1=affects others not at all to 5=affects others very much |
| Vulnerability (defecation related personal diarrhea risk) | If you defecate openly, how high is the risk to get diarrhea? | 1=not at all high to  5=very high |
| Vulnerability (defecation related diarrhea risk for community members) | If you defecate in the open, how high is the risk for ANY other community member to get diarrhea? | 1= not at all high to  5= very high |
| Health Knowledge |  | 1=no knowledge to 5=high knowledge |
| Feelings (proud) | How proud are you/ would you be of your own latrine? | 1= not proud at all to  5= very proud |
| Feelings (satisfaction) | How satisfied are you/ would you be with your own latrine? | 1= not proud at all to  5= very satisfied |
| Feelings (respect) | Do you think you are/ would be more respected by your community because you have a latrine? | 1= not proud at all to  5= very respected |
| Beliefs about costs and benefits (expensiveness) | Do you think that constructing a latrine is expensive? | 1= not at all expensive to  5= very expensive |
| Beliefs about costs and benefits (money, space, effort) | How difficult is it to find the money to build a latrine?  How difficult is it to find the appropriate space to build a latrine?  How difficult is it to find the time and effort to build a latrine? | 1= not at all difficult to  5= very difficult |
| Estimated number of other latrine owners (Others’ behavior/ relatives) | How many people of your relatives constructed a latrine? | 1=(almost) nobody to  5=almost all of them |
| Estimated number of other latrine owners (Others’ behavior/ community) | How many people of your community constructed a latrine? | 1=(almost) nobody to  5=almost all of them |
| Others’ (dis)approval (personal important people) | How much do people whose opinion about health is important to you approve or disapprove that you constructed a latrine for defecation? | 1=disapprove very much to  7=approve very much |
| Others’ (dis)approval (important people of the community) | People who are important in the community (e.g. religious leader, Chief of village, etc.) how much do they promote that you construct a latrine? | 1=not at all to  5=very much |
| Personal importance | Do you feel a personal obligation to construct a latrine? | 1=no obligation at all to  5=very strong obligation |
| Confidence in performance | How confident are you that you can construct a latrine even if this is difficult (e.g. because of flooding)? | 1=not at all confident to  5=very confident |
| Confidence in recovery | Imagine that the latrine got damaged. How confident are you to repair the latrine again? | 1=not at all confident to  5=very confident |
| Confidence in continuation | Imagine that it is the rainy season and danger of flooding. How confident are you that you can keep using your latrine? | 1=not at all confident to  5=very confident |
| How-to-do-knowledge |  | 1=no knowledge to 5=high knowledge |
| Commitment | Do you feel committed to having a latrine? | 1=not at all committed to  5=very committed |
| Communication | How often do you talk about latrine use with other people? | 1=not at all often to  5=very often |
