# Supplementary material for: Is Community-Led Total Sanitation connected to the rebuilding of latrines? Quantitative evidence from Mozambique
Source: PLoS One. 2018 May 22;13(5):e0197483. doi: 10.1371/journal.pone.0197483 (PMC5963780; doi:10.1371/journal.pone.0197483)
Supplement: S3 Table — (DOCX) [file pone.0197483.s003.docx]

**S3 Table**. **Predictors of Latrine Rebuilding in Logistic Regression Analysis.**

| **Modell** | ***B*** | ***SE*** | **Wald**  **X^2^ (1)** | **OR** | **95% CI** |
| --- | --- | --- | --- | --- | --- |
| **Modell 1: Personal and physical context factors** | | | | | |
| Age | .021 | .012 | 3.096^†^ | 1.02 | .99, 1.04 |
| Relationship status^a^ | .628 | .389 | 2.596 | 1.87 | .87, 4.01 |
| Years at school | .203 | .095 | 4.580* | 1.22 | 1.01, 1.47 |
| Able to read/ write^b^ | -.161 | .584 | .076 | .85 | .27, 2.67 |
| Religion: Catholic^c^ | -.505 | .309 | 2.676 | .60 | .329, 1.10 |
| Religion: Tribal and other^c^ | -.215 | .538 | .160 | .80 | .281, 2.31 |
| \| Household size \| \| --- \| | .153 | .080 | 3.668^†^ | 1.16 | .996, 1.36 |
| Average monthly income (MZN) of the family | .000 | .000 | .212 | 1.00 | 1.00, 1.00 |
| Risk of flooding | -.313 | .130 | 5.835* | .73 | .567, .943 |
| Soil condition: Clay^d^ | .570 | .318 | 3.208^†^ | 1.76 | .948, 3.29 |
| Soil condition: Rocky^d^ | -1.460 | .710 | 4.230* | .23 | .058, .93 |
| Distance to OD area | .723 | .142 | 25.899*** | 2.06 | 1.56, .27 |
| Constant | -3.437 | 1.622 | 4.492* | .03 |  |
| **Modell 2: Social context factors** | | | | | |
| Social dilemma | .472 | .129 | 13.293*** | 1.60 | 1.24, 2.07 |
| Social capital (solidarity) | .293 | .086 | 11.613** | 1.34 | 1.13, 1.59 |
| Social capital (trust) | .188 | .086 | 4.783* | 1.21 | 1.02, 1.43 |
| Social capital (empowerment and political action) | .153 | .107 | 2.061 | 1.17 | .95, 1.44 |
| Social capital (collective action and cooperation) | .090 | .146 | .381 | 1.09 | .82, 1.46 |
| Social capital (social cohesion and inclusion) | .250 | .100 | 6.210* | 1.28 | 1.06, 1.56 |
| Social identity (in-group ties) | .243 | .111 | 4.781* | 1.28 | 1.03, 1.59 |
| Social identity (centrality) | -.024 | .129 | .034 | .98 | .76, 1.26 |
| Social identity (in-group affects) | .047 | .097 | .235 | 1.05 | .87, 1.27 |
| Social cohesion (neighborhood cohesion) | -.223 | .125 | 3.187 | .80 | .63, 1.02 |
| Constant | -5.828 | 1.248 | 21.795*** |  |  |
| **Modell 3: RANAS factors** | | | | | |
| Vulnerability (personal general risk for diarrhea) | -.699 | .165 | 17.880*** | .50 | .36, .69 |
| Vulnerability (general health of community members) | .915 | .399 | 5.269* | 2.5 | 1.14, 5.45 |
| Vulnerability (defecation related personal diarrhea risk) | -.487 | .448 | 1.185 | .61 | .26, 1.48 |
| Vulnerability (defecation related diarrhea risk for community members) | .344 | .458 | .564 | 1.41 | .58, 3.47 |
| Health Knowledge | -.434 | .278 | 2.433 | .65 | .38, 1.12 |
| Feelings (proud) | .570 | .360 | 2.508 | 1.77 | .87, 3.58 |
| Feelings (satisfaction) | .053 | .419 | .016 | 1.06 | .46, 2.40 |
| Feelings (respect) | -.297 | .190 | 2.447 | .74 | .51, 1.08 |
| Beliefs about costs and benefits (expansiveness) | -.330 | .179 | 3.389 | .72 | .51, 1.02 |
| Beliefs about costs and benefits (money, space, time) | -.558 | .453 | 1.515 | .57 | .24, 1.39 |
| Estimated number of other latrine owners (Others’ behavior/ relatives) | .296 | .196 | 2.277 | 1.34 | .92, 1.97 |
| Estimated number of other latrine owners (Others’ behavior/ community members) | 1.067 | .234 | 20.844*** | 2.91 | 1.84, 4.60 |
| Others’ (dis)approval (personal important others’) | .369 | .224 | 2.714 | 1.45 | .93, 2.24 |
| Others’ (dis)approval (important persons of the community) | -.363 | .222 | 2.671 | .70 | .45, 1.08 |
| Personal importance | -.149 | .136 | 1.189 | .86 | .66, 1.13 |
| Confidence in performance | .244 | .194 | 1.586 | 1.28 | .87, 1.87 |
| Confidence in recovery | .661 | .281 | 5.527* | 1.94 | 1.12, 3.36 |
| Confidence in continuation | -.206 | .201 | 1.059 | .81 | .55, 1.21 |
| How-to-do-knowledge | .185 | .256 | .518 | 1.20 | .73, 1.99 |
| Commitment | -.010 | .138 | .006 | .99 | .76, 1.30 |
| Communication | .409 | .183 | 5.021* | 1.51 | 1.05, 2.15 |
| Constant | -9.835 | 2.762 | 12.683 |  |  |
| **Modell 4: significant context and RANAS factors from model 1+2+3** | | | | | |
| Relationship status^a^ | .741 | .501 | 2.189 | 2.09 | .78, 5.59 |
| Years at school | .250 | .089 | 7.846** | 1.28 | 1.07, 1.53 |
| Risk of flooding | -.099 | .168 | .344 | .90 | .65, 1.26 |
| Soil condition: Clay^d^ | .873 | .436 | 4.014* | 2.39 | 1.01, 5.62 |
| Soil condition: Rocky^d^ | -1.600 | .881 | 3.299^†^ | .20 | .03, 1.13 |
| Social dilemma | .039 | .177 | .048 | 1.03 | .73, 1.46 |
| Social capital (solidarity) | .136 | .127 | 1.158 | 1.14 | .89, 1.46 |
| Social capital (trust) | -.044 | .140 | .098 | .95 | .72, 1.25 |
| Social capital (social cohesion and inclusion) | .445 | .160 | 7.753** | 1.56 | 1.14, 2.13 |
| Social identity (in-group ties) | .101 | .143 | .503 | 1.10 | .83, 1.46 |
| Vulnerability (personal general risk for diarrhea) | -.611 | .155 | 15.508*** | .54 | .40, .73 |
| Vulnerability (general health of community members) | .657 | .340 | 3.735^†^ | 1.92 | .99, 3.75 |
| Estimated number of other latrine owners (Others’ behavior/ community members) | 1.106 | .221 | 24.938*** | 3.02 | 1.95, 4.66 |
| Confidence in recovery | .910 | .271 | 11.321** | 2.48 | 1.46, 4.22 |
| Communication | .118 | .201 | .343 | 1.12 | .75, 1.66 |
| Distance to OD area | .265 | .180 | 2.163 | 1.30 | .91, 1.85 |
| Constant | -13.670 | 2.591 | 27.834*** | .00 |  |

*Note.* *N* =278 . For the model of personal and physical context factors (Model 1) R^2^= .35 (Nagelkerke). X^2^(12) =, p < .0005. For the model of social context factors (Model 2) R^2^=.27. (Nagelkerke). X^2^(10) =, p < .0005. For the model of psychosocial factors (Model 3) R^2^=.68 (Nagelkerke). X^2^(21) =, p < .0005. For the overall model of significant context and psychosocial factors (Model 4) R^2^=.68 (Nagelkerke). X^2^(16) =, p < .0005.
^a^ no relationship as reference category; ^b^ not able to read/ write as reference category; ^c^ Muslim as reference category; ^d^ sandy as reference category
OR = odds ratio; CI = confidence interval; **P* < .05; ***P* < .005; ****P* < .0005; † P <.10
